# Different Cytotoxicity Induced by Hexabromocyclododecanes on Mouse Neuroblastoma N2a Cells via Oxidative Stress and Mitochondrial Apoptotic Pathway

**DOI:** 10.3390/toxics12090665

**Published:** 2024-09-12

**Authors:** Keyan Wan, Dongting Wu, Guangshan Xie, Yunxiu Li, Jianqing Zhang

**Affiliations:** 1 Shenzhen Bao’an District Songgang People’s Hospital, Shenzhen 518105, China; 2 Department of POPs Lab, Shenzhen Center for Disease Control and Prevention, Shenzhen 518055, China; 3 Shenzhen Eye Hospital, Jinan University, Shenzhen Eye Institute, Shenzhen 518010, China

**Keywords:** HBCDs, oxidative damage, mitochondrial pathway

## Abstract

Hexabromocyclododecane (HBCD) is widely used in polystyrene foams, building materials, and electrical equipment as a brominated flame retardant (BFR) and persists in the environment and human body matrix. It has attracted increased attention since its neuroendocrine disorder effects have been observed in humans and animals. However, studies evaluating the neurotoxicity of HBCD diastereoisomers and the potential mechanisms involved are still limited. In this study, we compared the cytotoxicity induced by the three HBCD diastereoisomers (i.e., α-, β-, and γ-HBCD) in N2a cells and further investigated the underlying molecular mechanism. Our results showed that HBCD diastereoisomers decreased cell viability in the order of β-HBCD > α-HBCD > γ-HBCD. Moreover, α-HBCD and β-HBCD exposure led to different degrees of cell cycle disruption and oxidative stress of N2a cells, implying that oxidative stress-mediated differential cytotoxicity of HBCD diastereoisomers. The expressions of caspases and Bcl-2 were differentially regulated by α-HBCD and β-HBCD, suggesting that the mitochondrial apoptosis pathway may be critical in HBCDs-mediated N2a cell toxicity. Therefore, our studies provided novel evidence for the underlying mechanisms of the distinct cytotoxicity of HBCD diastereoisomers.

## 1. Introduction

**Figure 1:**
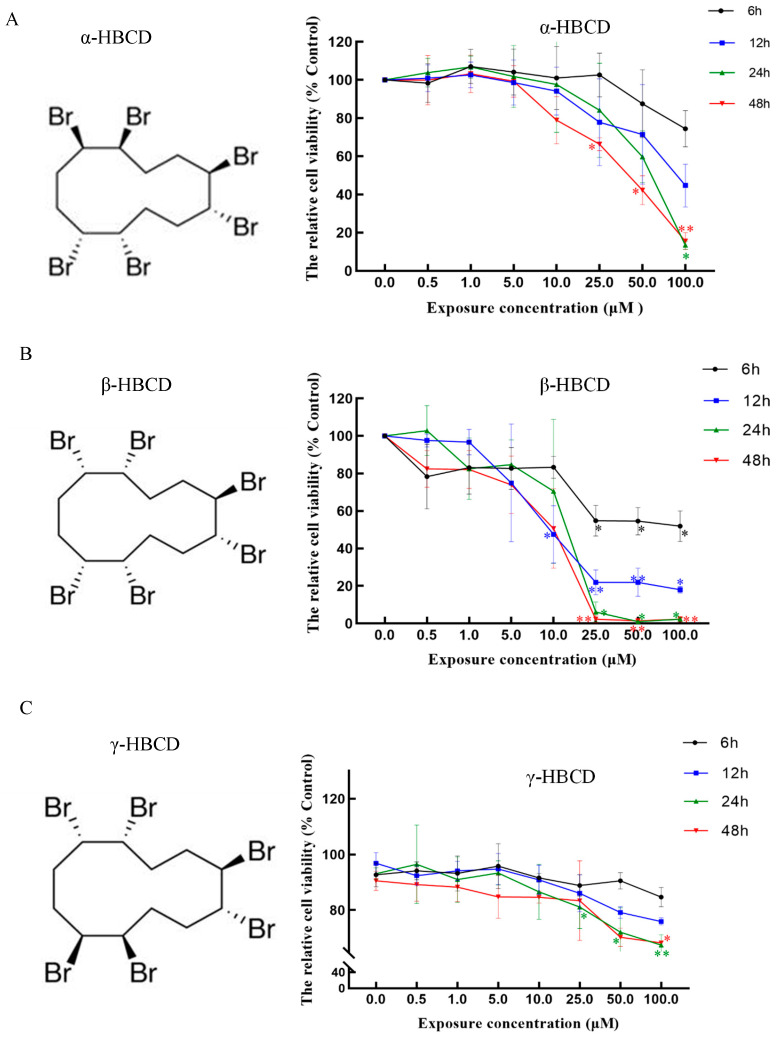
The viability of N2a cells exposed to α-HBCD (**A**), β-HBCD (**B**), and γ-HBCD (**C**) on the right panels. The chemical structure of each stereoisomer of HBCD is shown in the left panels. ** *p* < 0.01, * *p* < 0.05 versus the control.

1,2,5,6,9,10-hexabromocyclododecane (HBCD), as an additive brominated flame retardant (BFR), has been widely used in polystyrene foams, building materials, upholstery textiles, and electrical equipment as a substitute for PBDEs. Due to ubiquitous contamination, long-range transportation, high bioaccumulation, and risk to human health, HBCDs were listed in the Stockholm Convention 2013 [1]. Although HBCDs comprise 16 diastereomers, technical products primarily include three isomers (α-, β-, and γ-HBCD), of which the predominant is γ-HBCD followed by α- and β-HBCD, accounting for 75–89%, 1–13%, and 1–12%, respectively [2]. The differences between the three isomers of HBCD are mainly due to the different positions and spatial arrangements of the bromine atoms, as shown in Figure 1. γ-HBCD is generally reported to be the most abundant isomer in commercial mixtures and environmental samples. However, α-HBCD is most abundant in biota and human beings, possibly because of the metabolic capability of distinct organisms and the different environmental behaviors of the isomers [3]. Animal studies have shown that commercial HBCDs is mainly metabolized by the cytochrome P450 enzymes via oxidative and reductive debromination [4,5,6], while the stereoselective metabolism of HBCD diastereoisomers by cytochrome P450 3A4 (CYP3A4) enzyme was demonstrated in a previous study, which may contribute to the different abundances of HBCD diastereoisomers in organisms [7]. Consistent with this finding, the metabolism of HBCD diastereomers was founded to follow the order ß-HBCD > γ-HBCD > α-HBCD in male rats, due to the more extensive metabolic pathways of ß-HBCD and γ-HBCD compared to α-HBCD, such as stereoisomerization, oxidation, dehydrogenation, reductive debromination, and ring opening [8]. There has been extensive literature on the levels, characterization, migration, and transport of HBCDs in the environment and food chain, and even in human biological samples. However, studies on the toxicity and the underlying mechanism of HBCDs are still limited.

As an endocrine-disrupting chemical, HBCDs induce thyroid hormone disruption and neurobehavioral and developmental disorders, and their exposure has been linked to a variety of diseases, including the reproductive, immune, and cardiovascular systems. Specifically, HBCD exposure was found to decrease total thyroxine (T4) levels and increase pituitary and thyroid weights, thyroid stimulating hormone (TSH) in the pituitary, and thyroid follicular cell activation in female rats [9,10]. Previous studies have implicated the toxic potential of HBCD to the thyroid in rats as well as in human cells by disrupting thyroid hormone receptor-mediated gene expression [3,11,12,13]. The estrogenic effects of HBCD as estrogen receptor (ER) targets in the corresponding tissues have been elucidated, leading to the activation of transcription factors that activate certain genes and cell-specific responses [14,15]. As a result, thyroid hormones also have an impact on other systems, including the liver, nervous system, immune, cardiovascular system, and metabolism [16]. HBCD was found to affect IFN-γ secretion in various immune cells through the activation of the p44/42 pathway, causing inappropriate inflammation [17]. In vivo and in vitro studies showed that HBCD affects the cardiovascular system by inducing cardiac hypertrophy and arrhythmias [18,19]. Furthermore, HBCD has been shown to induce hepatic cell injury by stimulating the activation of the PI3K/Akt pathway and AMPK and p38 MAPK signaling [20]. HBCD stereoisomers exposure to infant mice lead to the disruption of metabolic pathways, including glycolysis, lipid metabolism, and the citric acid cycle [21]. Notably, HBCD can cause neuronal cell death [22,23,24] and harm brain development, leading to neurobehavioral dysfunction and compromised learning and memory abilities [25]. It is well known that the neuroendocrine system is essential in mediating neurodevelopment and maintaining normal endocrine function in organisms [24], and a disturbance in this system potentially causes neural disorders or neurotoxic effects on humans [26,27]. Given the extensive multisystemic adverse effects of HBCDs and the essential role of neurological damage involved, it is necessary to evaluate and elucidate the molecular mechanisms involved in HBCDs-induced neurotoxicity.

Extensive studies have shown that different HBCD diastereoisomers exhibit different toxicological risks in virous models. HBCD diastereoisomers showed different cytotoxicity mainly due to differential degradation rate and different binding modes with hydrocarbon receptors (AHRs) in vivo and in vitro studies [21,28,29,30]. Due to its high lipophilicity, HBCD is capable of penetrating the blood–brain barrier and accumulating in the brain because of its high lipophilicity (log Kow 5.6), potentially leading to neurotoxic effects by perturbing nervous system pathways and functions [21,31,32]. Although the neurotoxicity of HBCDs has been reported, the diastereoisomer-specific neurotoxicity of HBCD remains unclear. As evidence for the different toxicological risks induced by HBCD diastereoisomers and the critical role of neurological damage in HBCD-induced multisystem damage, it is imperative to evaluate the neurotoxicity of different HBCD diastereoisomers and explore its underlying mechanisms, contributing to a better understanding HBCD-induced neuronal impairment as an endocrine-disrupting chemical and facilitating the precise prevention and possible treatment of HBCD neurotoxicity.

This study determined and compared the cytotoxicity, including the oxidative damage, cell cycles, and apoptosis of three HBCD isomers, in mouse neuroblastoma N2a cells. The mRNA and protein expression levels of several apoptosis-related proteins, including caspase-3 and Bcl-2, were further measured to explore their potential molecular mechanisms from the perspective of mitochondrial apoptosis pathways. The results will provide more comprehensive evidence on the underlying mechanism of cytotoxicity effects induced by HBCD isomers.

## 2. Materials and Methods

### 2.1. Reagents

(±) α-hexabromocyclododecane (α-HBCD) (≥99.5%), (±) β-hexabromocy clododecane (β-HBCD) (≥97.0%), (±) γ-hexabromocyclododecane (γ-HBCD) (≥99.0%), and dimethyl sulfoxide (DMSO) were purchased from Sigma-Aldrich (St. Louis, MO, USA). RPMI-1640 medium, fetal bovine serum (FBS), trypsin, penicillin, and streptomycin were from Gibco (Invitrogen, Paisley, UK). Cell Counting Kit-8 (CCK-8) were from Dojindo Chemical Laboratory (Kumamoto, Japan), the Glutathione Peroxidase (GSH-Px) Assay Kit, malondialdehyde (MDA) Assay Kit, Cycletest™ Plus DNA Reagent Kit (BD Biosciences, San Jose, CA, USA), RNeasy Plus Mini Kit (Qiagen, Hilden, Germany), Prime Script™ 1st Strand cDNA Synthesis Kit, SYBR^®^ Premix Ex TaqTM (Perfect Real Time, TAKARA, Tokyo, Japan), and polyvinylidene fluoride membrane kit were purchased from Millipore (Darmstadt, Mullica, NJ, USA). The Bicinchoninic Acid (BCA) Protein Assay Kit and chemiluminescence and Western Blotting Substrates were purchased from Thermo Fisher Scientific (Waltham, MA, USA). The SeeBlue^®^ Pre-Stained Standard was purchased from Invitrogen (Invitrogen, Carlsbad, CA, USA). The following antibodies were purchased: anti-murine 8-oxoguanine DNA glycosylase (mogg1, PA1-16505; Thermo Fisher Scientific); caspase-3, cleaved caspase-3 (Asp175), cleaved caspase-9 (Asp353), and B-cell lymphoma 2 (Bcl-2) (D17C4) rabbit monoclonal (mouse preferred; Cell Signaling Technology, Beverly, MA, USA); caspase-9 (EPR 18107; and horseradish peroxidase (HRP)-labeled goat anti-mouse secondary antibody (Millipore). The other antibodies were analytical-grade chemicals.

### 2.2. Cell Culture

The N2a cell line was provided by the Cell Bank of Chinese Academy of Sciences, and cultured in RPMI-1640 medium (Invitrogen, Paisley, UK) supplemented with 10% FBS, 100 U/mL (1%) penicillin, and 100 U/mL (1%) streptomycin under a 5% CO_2_ humidified atmosphere at 37 °C.

### 2.3. Cell Viability Assay

The cells were seeded in a 96-well plate at 1 × 10^4^ cells/well, with 100 μL per well, and cultured for 36 h. When the cell fusion reached 90%, a series of subsequent experiments were conducted. The α-HBCD, β-HBCD, and γ-HBCD standard were added to 1 mL sterile DMSO to make 25 µg/μL stock solution and were stored at 4 °C. This was diluted with serum-free RPMI-1640 medium to different concentrations (0.5, 1, 5, 10, 25, 50, 100 μmol/L) before infection. A total of 100 μL of cell suspension was added to each well of a 96-well plate and was cultured for 24 h. Then, 10 μL of different concentrations of alpha-, beta-, and gamma HBCD were added. An amount of 0.5% DMSO was used as the solvent control group and was cultured for 6, 12, 24, and 48 h before measurement. When the exposure was terminated, the medium was carefully discarded from each well, and the cell viability was measured by the CCK-8 assay. Namely, 10 μL CCK-8 reagent together with 100 μL RPMI-1640 was added per well, and the plate was incubated at 37 °C for 2 h. The absorbance (λ = 450 nm) was measured using the Infinite Pro M1000 Multi Detection Plate Reader (Infinite^®^M1000; Tecan, Morrisville, NC, USA). Five parallel wells were designed for each exposure condition, and the assay was repeated three times. The relative cell viabilities were finally evaluated by dividing the absorbance values in exposure groups by those of the vehicle control.

### 2.4. Experiments for Oxidative Stress

The N2a cells were seeded in 6-well plates at a density of 1 × 10^6^ cells/mL. After 36 h culture, cells were treated with different concentrations of α-HBCD (1.0, 10.0, and 50.0 μM) and β-HBCD (1.0, 5.0, and 10.0 μM) for 24 h. After exposure, the protein sample was harvested from each well and submitted for analysis of MDA and GSH-Px activity using commercial MDA and GSH-PX assay kits (BD Biosciences), respectively. After treatment, the cells were incubated with 10 mM DCFH-DA at 37 °C for 30 min in the dark, and the fluorescence was measured at excitation/emission wavelengths of 498/525 nm, respectively. After treating the cells in the same way as described above, H_2_O_2_ was prepared at a ratio of 1:1000, and prepared H_2_O_2_ (20 μmol/L) was added 30 min before cell collection as a positive control group. Intracellular reactive oxygen species (ROS) generation was assessed using the DCFH-DA (2,7-dichlorofuorescin diacetate) fluorescent probe. DCFH-DA working solution (10 μmol/L) was prepared by diluting DCFH-DA stock solution (10 mM) with serum-free culture medium at a ratio of 1000:1. A total of 1 mL of pre-warmed working solution was added to each sample and resuspended. The EP tubes were incubated in an incubator at 37 °C for 30 min in the dark, and the tubes were mixed by inverting them every 3–5 min. The cells were centrifuged at 1000 rpm × 3 min and washed three times with warm PBS at 1000 rpm × 3 min, and care was taken to discard the supernatant to remove the DCFH-DA background. The collected cells were resuspended in 1 mL of PBS; the fluorescence was measured at excitation/emission wavelengths of 498/525 nm, respectively; and the average OD value measured by the zymograph was calculated. The experiment was repeated three times, and all measured ROS values were normalized by the corresponding viability in this study.

### 2.5. Cell Cycle Analysis

The N2a cells were treated similarly to the protocol used for oxidative stress experiments. After treatment, cells were trypsinized, harvested, and washed twice with 1 mL of PBS (phosphate-buffered saline, pH 7.4) buffer solution. The resuspended cell suspension from each group was adjusted to a density of 1.0 × 106 cells/mL. Cells were analyzed using the Cycletest™ Plus DNA Reagent Kit according to the manufacturer’s instructions (BD Biosciences). Cell cycle distribution was detected by flow cytometry (FACS CantoII Cell Analyzer; BD Biosciences).

### 2.6. Quantitative Polymerase Chain Reaction

The N2a cells were seeded in 25 cm^2^ cell culture flasks at a density of 1.0 × 10^6^ cells/mL and treated with 0, 1.0, 10.0, and 50.0 μM α-HBCD and 0, 1.0, 5.0, and 10.0 μM β-HBCD for 24 h. After the termination of the treatment, total RNA was extracted from the cell samples using the RNeasy Plus Mini Kit (Qiagen). Namely, the samples were treated with deoxyribonuclease I to remove the contamination of genomic DNA, and first-strand cDNA was synthesized using the Prime Script™ RT Reagent Kit (TaKaRa, Tokyo, Japan) from 400 ng RNA of each sample. All cDNA samples were subsequently submitted to quantitative PCR (qPCR) analysis with the MX4000 Real-Time qPCR System (Stratagene, La Jolla, CA, USA) using SYBR Premix Ex Taq^TM^ (Tli RNase H Plus) (TaKaRa, Tokyo, Japan), and the target genes included caspase-3, caspase-9, and Bcl-2. The primer sequences were designed by Primer 5.0 and synthesized by Sangon Biotech Co., Ltd. (Shanghai, China). The GAPDH housekeeping gene was used as an internal control to normalize the expression levels of the target genes. The final transcriptional levels of the target genes were calculated using the ΔΔCT method. The primer sequences for each mRNA are shown in Table 1.

### 2.7. Western Blot

Total protein was extracted from the cell samples described above using lysis buffer containing 1% NP-40, 1% sodium dodecyl sulfate, 0.5% sodium deoxycholate, 10 μg/mL phenylmethylsulfonyl fluoride, 0.2 mM ethylenediaminetetraacetic acid, and 0.5 mM DL-dithiothreitol. A total of 250 μL of lysis buffer was added into a 25 cm^2^ culture flask and centrifuged at 13,000× *g* for 30 min at 4 °C, and the supernatant was collected for protein concentration measurement by the BCA method. The collected cells were centrifuged at 1000 rpm for 5 min, and the supernatant was aspirated. The cells were washed 1–2 times with 1 × PBS at 1000 rpm for 3 min, and a certain amount of lysis buffer was added. The cells were lysed for 30 min and then aspirated for testing. The BCA working solution was prepared according to the instructions of the BCA kit and was incubated at 37 °C for 15 min. A total of 25 μL of 2,4-dinitrophenylhydrazine was added to each well and was mixed well. After 15 min of incubation at 37 °C, 250 L of 0.4 mol/L NaOH solution was added to each well and mixed well. After 5 min, the absorbance was measured at 450 nm on an enzyme meter.

An aliquot of protein (20 μg) was separated on 12.5% SDS-PAGE gels and electrotransferred to the PVDF membrane. The membrane was blocked in 5% bovine serum albumin for 1 h at room temperature and then incubated with mouse anti-mOGG1 primary antibody (Santa Cruz Biotechnology, Dallas, TX, USA) or mouse anti-β-actin antibody (Santa Cruz Biotechnology) overnight at 4 °C, which was further incubated with the corresponding HRP-conjugated secondary antibody for 1 h at room temperature. The concentration of mouse anti-mOGG1 primary antibody is 1:1000, and the concentration of secondary antibody is 1:4000. The membrane was scanned using the ImageQuant RT ECL Imager after reacting with a chemiluminescence substrate (Pierce, Rockford, IL, USA). The relative level of each protein to β-actin was determined by densitometry analysis using Image J 2.0 software (National Institutes of Health, Bethesda, MD, USA). N2a cell samples with 24 h α-HBCD and β-HBCD treatments were also analyzed by immunoblotting for cleaved caspase-3, caspase-3, cleaved caspase-9, caspase-9, and Bcl-2 expression with the corresponding antibodies (cleaved caspase-3 and cleaved caspase-9; all provided by Santa Cruz). Caspase-3, caspase-9, Bcl-2 (provided by Abcam, Cambridge, MA, USA), and β-actin were used as the internal reference.

Whole-cell lysates were extracted using lysis buffer containing 1% NP-40, 1% sodium dodecyl sulfate, 0.5% sodium deoxycholate, 10 μg/mL phenylmethylsulfonyl fluoride, 0.2 mM ethylenediaminetetraacetic acid, and 0.5 mM DL-dithiothreitol. A total of 250 μL of lysis buffer was added into a 25 cm^2^ culture flask and was centrifuged at 13,000× *g* for 30 min at 4 °C; the milky supernatant was harvested and the insoluble debris was discarded. The supernatant was collected for the detection of protein concentration by the BCA method.

### 2.8. Statistical Analysis

Each experiment was independently performed three times or more. The data are presented as the mean ± standard deviation (SD). Differences were considered statistically significant at *p* < 0.05 using one-way analysis of variance and the two-tailed Student’s *t*-test (SPSS 22.0; Chicago, IL, USA).

## 3. Results

### 3.1. Effects of HBCD on Cell Viability

The effects of three diastereomers of HBCDs on the viability of N2a cells are shown in Figure 1. The results showed that the viability of N2a cells exposed to α-HBCD and β-HBCD was reduced in the exposure dose range of 0.5–100 μmol/L from 6 to 48 h, which showed that viability significantly declined in a time- and concentration-dependent manners (Figure 1A,B; *p* < 0.05). However, there was no significant difference in cell viability between 24 and 48 h exposure (*p* > 0.05). Therefore, we chose 24 h as the appropriate exposure time for subsequent experiments. When the cells were exposed to α-HBCD concentrations of 1.0, 25.0, and 50.0 μmol/L for 24 h, the cell viabilities were 103.3 ± 0.7%, 67.2 ± 2.4%, and 57.1 ± 1.2% of the control, respectively (Figure 1A; *p* < 0.05). When the cells were exposed to 10 μM α-HBCD for 6, 12, 24, and 48 h, the cell viability was 91.9 ± 0.9%, 80.5 ± 3.0%, 69.8 ± 2.4%, and 72.3 ± 0.7% of the control, respectively. When the cells were exposed to β-HBCD concentrations of 1.0, 5.0, and 25.0 μmol/L for 24 h, the cell viabilities were 83.3 ± 4.2%, 84.6 ± 4.4%, and 6.0 ± 0.6% of the control (Figure 1B; *p* < 0.05). Overall, β-HBCD had a more obvious inhibitory effect on cell viability. However, γ-HBCD exerted much less obvious inhibitory effects on cell growth, compared with the other two diastereomers (Figure 1C). Furthermore, the 24 h exposure IC_50_ values of α-HBCD and β-HBCD were calculated to be 60.1 and 10.5 μM, respectively, for N2a cells according to the cell viability curve. Apparently, β-HBCD showed the highest toxicity to N2a cells. To further investigate the toxicological mechanisms of HBCDs, we set the doses of 1.0, 10.0, and 50.0 μM for α-HBCD and 1.0, 5.0, and 10.0 μM for β-HBCD, representative of low, medium, and high exposure doses for further study.

### 3.2. Oxidative Damage Induced by HBCDs in N2a Cells

**Figure 2:**
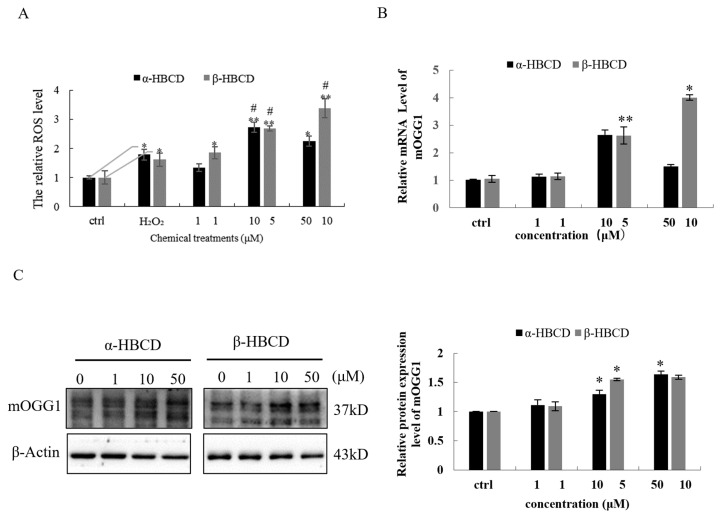
Oxidative stress determination in N2a cells induced by α-HBCD and β-HBCD treatment. (**A**) ROS generation. (**B**) Transcription levels of mOGG1. (**C**) Western blotting for mOGG1 and the quantification results are shown in the right panel. Note: ** *p* < 0.01; * *p* < 0.05 versus the control. ^#^ *p* < 0.05 versus the H_2_O_2_ positive control group.

GSH-PX activity was detected. As shown in Table 2, the content of MDA in N2a cells significantly increased in the medium- and high-dose groups after exposure to both α-HBCD and β-HBCD (*p* < 0.05). In contrast, the activity of GSH-PX significantly decreased in a concentration-dependent manner for both α-HBCD and β-HBCD (*p* < 0.05). Furthermore, ROS levels in N2a were further detected, as shown in Figure 2A. ROS levels in N2a cells in the medium- and high-concentration groups of α-HBCD and the three groups of β-HBCD were significantly higher than those of the control group (*p* < 0.05). ROS levels in β-HBCD-treated groups were elevated in a dose-dependent manner, whereas α-HBCD did not show a dose-dependent manner. In addition, the ROS level of the middle-dose α-HBCD group was 1.5 times that of the H_2_O_2_-positive control group (*p* < 0.05). The ROS level of the high-dose group was slightly higher than that of the H_2_O_2_ group, and the difference was not statistically significant. The oxidative damage ability of the low-dose β-HBCD group was slightly higher than that of the 20 μmol/L H_2_O_2_-positive control group. Meanwhile, the ROS levels induced by β-HBCD in the middle- and high-dose groups were 1.7- and 2.1-fold that of the hydrogen peroxide treatment group, respectively (*p* < 0.05).

In our study, the levels of MDA in N2a cells were significantly increased by 1.7- and 2.2-fold, and the levels of ROS were significantly increased by 2.7- and 2.2-fold following medium- and high-dose α-HBCD exposure for 24 h (*p* < 0.05). Similarly, the levels of MDA in N2a cells were significantly increased by 2.1- and 2.6-fold, and the levels of ROS were significantly increased by 2.7- and 3.4-fold following medium- and high-dose β-HBCD exposure for 24 h (*p* < 0.05). The production of ROS positively correlated with MDA content in N2a cells, and the correlation coefficient was 0.73 in the α-HBCD treatment group (*p* < 0.01) and 0.67 in the β-HBCD treatment group (*p* < 0.05).

The mRNA and protein expression levels of mOGG1 were investigated in this study. The expression of mOGG1 was upregulated in a dose-dependent manner in N2a cells after exposure to β-HBCD, whereas mOGG1 was not upregulated in a dose-dependent manner in the α-HBCD-treated group (Figure 2B,C). The mRNA expression of mOGG1 was highest in the medium-dose group of α-HBCD, which was increased to 2.9-fold of the negative control group. The mRNA expression level of mOGG1 in N2a cells was increased to 1.1- and 1.4-fold in the low- and high-dose groups of α-HBCD, respectively. Furthermore, mOGG1 protein was increased to 1.1-, 1.3- (*p* < 0.05), and 1.6-fold (*p* < 0.05) in N2a cells incubated with 1.0, 10.0, and 50.0 μM α-HBCD compared with the negative control, respectively. Moreover, for β-HBCD, the mRNA expression of mOGG1 was increased to 1.1-, 2.5-, and 3.8-fold compared with the negative control group at concentrations of 1.0, 5.0, and 10.0 μM, and the expression difference was significant in the medium- and high-dose group (*p* < 0.05). Similarly, the protein expression level of mOGG1 was increased to 1.1-, 1.3- (*p* < 0.05), and 1.7-fold compared with the negative control after N2a cells were exposed to β-HBCD (*p* < 0.05).

### 3.3. Effects of α-HBCD and β-HBCD on the Cell Cycle of N2a Cells

As shown in Table 3, more cells in the medium-dose group of α-HBCD were blocked in the G0/G1 phase than in the control group (*p* < 0.01), and the number of cells in the S phase was significantly less than that of the control group (*p* < 0.05). The results showed that the rate of DNA synthesis decreased and cell proliferation was inhibited in the middle-dose group of α-HBCD. At the high concentration of α-HBCD (50.0 μM), the proportion of cells in the G2/M and (G2 + S) phases increased (*p* < 0.01), which indicated that cell proliferation was promoted. However, with the increasing concentrations of β-HBCD, the number of cells in the G1 phase decreased and the number of cells in the G2/M phase significantly increased (*p* < 0.01). Although the number of cells in the S phase was significantly less than that in the control group in the medium-dose β-HBCD group, as the concentration of HBCD increased, the G2/M and (G2 + S) phase cells increased (*p* < 0.01), indicating that the cells were arrested in the G2/M phase. Thus, the increasing dose of α-HBCD and β-HBCD gradually decreased the number of cells in the G0/G1 phase. DNA synthesis was limited, and the cells were arrested in the G2/M phase, and finally, the proliferative activity of cells was inhibited. In summary, the cell proliferation was inhibited and the cell cycle was arrested at the G2/M phase following the exposure of the N2a cells to α-HBCD and β-HBCD.

### 3.4. Effects of α-HBCD and β-HBCD on Mitochondria Function of N2a Cells

**Figure 3:**
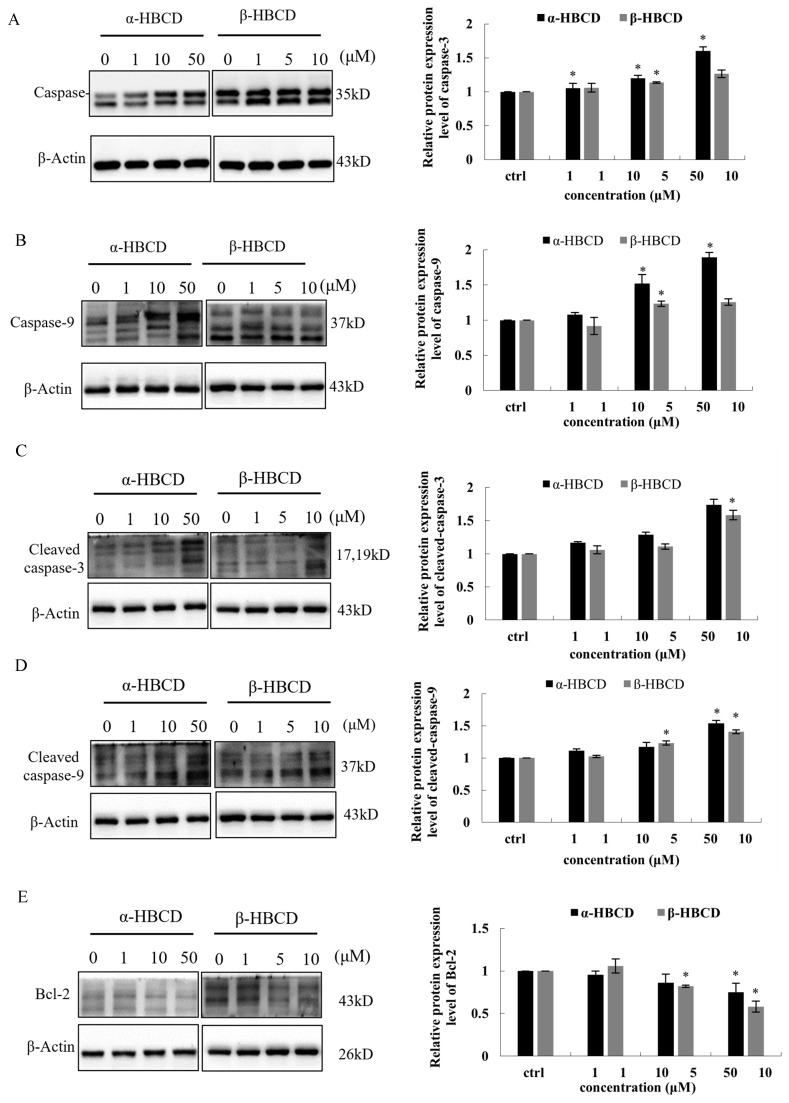

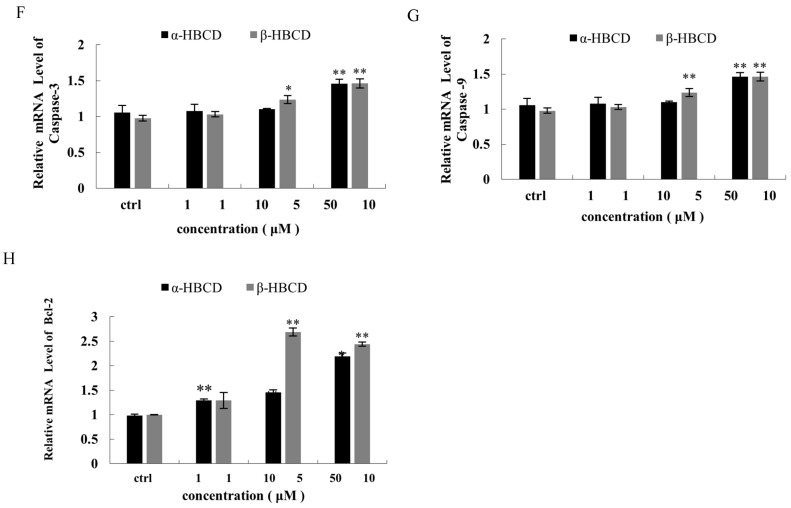
Caspase activation in N2a cells exposed to α-HBCD and β-HBCD treatment. The protein expression of caspase-3, caspase-9, bcl-2, cleaved caspase-3, and cleaved caspase-9 is shown in (**A**–**E**), respectively. The mRNA levels of caspase-3, caspase-9, and Bcl-2 are shown in (**F**–**H**), respectively. Data are expressed as the mean ± SD (*n* = 3). ** Significant difference from control (*p* < 0.01); * Significant difference from control (*p* < 0.05).

Furthermore, the expressions of caspase-3, caspase-9, cleaved caspase-3, cleaved caspase-9, and Bcl-2 were fully investigated in N2a cells exposed to α-HBCD and β-HBCD. The results are presented in Figure 3. Caspase-3, caspase-9, cleaved caspase-3, and cleaved caspase-9 were upregulated in N2a cells treated with both α-HBCD and β-HBCD. The protein expression of caspase-3 in N2a cells incubated with α-HBCDs (1.0, 10.0, and 50.0 μM) for 24 h were increased to 1.1-, 1.2-, and 1.6-fold (*p* < 0.05) of the vehicle control (Figure 3A), and the mRNA levels were elevated to 1.0-,1.0-, and 1.4-times (*p* < 0.05) of the control group (Figure 3F). Similarly, the levels of mRNA and protein caspase-3 in N2a cells were increased to 1.1-, 1.3- (*p* < 0.05), and 1.5- (*p* < 0.05) and 1.1-, 1.1- (*p* < 0.05), and 1.3-fold compared with the negative control group at concentrations of low-, medium-, and high-dose group of β-HBCD, respectively (Figure 3A,F; *p* < 0.05). The mRNA and protein levels of caspase-9 in N2a cells were increased to 1.1-, 1.2-, and 1.7-fold and 1.1-, 1.5- (*p* < 0.05), and 1.9-fold (*p* < 0.05) compared with the negative control group in the low-, medium-, and high-dose groups of α-HBCD, respectively (Figure 3B,G *p* < 0.05). Similarly, the mRNA and protein expression of caspase-9 in N2a cells was increased to 1.0-, 1.6- (*p* < 0.05), and 1.7-fold (*p* < 0.05) and 0.9-, 1.2- (*p* < 0.05), and 1.3-fold compared with the negative control group in the low-, medium-, and high-dose groups of β-HBCD, respectively (Figure 3B,G, *p* < 0.05). The level of Bcl-2 protein in N2a cells was decreased by 4%, 14%, and 25% (*p* < 0.05) and by -6%, 18% (*p* < 0.05), and 42% (*p* < 0.05) in response to increasing concentrations of α-HBCD and β-HBCD, respectively (Figure 3C). However, the mRNA expression of Bcl-2 was increased to 1.3-, 1.5-, and 2.2-fold (*p* < 0.05) and 1.3-, 2.7- (*p* < 0.05), and 2.4-fold (*p* < 0.05) in response to increasing concentrations of α-HBCD and β-HBCD, respectively (Figure 3H). Meanwhile, the protein level of cleaved caspase-3 and cleaved caspase-9 was increased to 1.2-, 1.3-, and 1.7-fold and 1.1-, 1.2-, and 1.5-fold (*p* < 0.05) compared with the negative control group in the low-, medium-, and high-dose groups of α-HBCD, respectively (Figure 3D,E, *p* < 0.05). Similarly, the protein level of cleaved caspase-3 and cleaved caspase-9 was increased to 1.1-, 1.1-, and 1.6-fold (*p* < 0.05) and 1.0-, 1.2- (*p* < 0.05), and 1.4-fold (*p* < 0.05) compared with the negative control group in the low-, medium-, and high-dose groups of β-HBCD, respectively (Figure 3D,E).

## 4. Discussion

The high frequency of detection and different abundances of HBCD diastereoisomers in various environmental, biological matrices, and human tissues—in particular, the most-studied α-, β-, and γ-HBCD—have led to an increased attention to this type of endocrine disruptor [33,34,35]. Exposure to HBCD diastereoisomers has been shown to have different toxicity from in vitro and in vivo studies because of the different lipophilicity [21], metabolic rate [8] and binding affinities to respective receptors [30]. HBCD has been reported to disrupt the nigrostriatal dopamine system, which may increase the risk of Parkinson’s disease [32]. Thyroid hormones are essential for neural stem cell differentiation and brain development, and their deficiency can result in irreversible neurological effects. Correspondingly, neuronal cells are classical target cells for thyroid hormone actions, especially during development [36]. Thus, it is necessary to explore their neurotoxicity and the underlying mechanisms that contribute to the precise prevention and control of these endocrine disruptors. HBCD was found to damage dopamine neurons and cell viability in different neuronal cell models [24,32]. However, reports on the toxic effects of HBCD isomers in neuronal cells are scarce, and in particular, the underlying mechanisms have not been adequately explained. In this study, mouse neuroblastoma N2a cell line was used as a model to investigate the toxic effects of three HBCD diastereoisomers. Our results showed that HBCD diastereoisomers differentially reduced cell viability and followed the order of β-HBCD > γ-HBCD > α-HBCD. Moreover, the different effects of β-HBCD and γ-HBCD in the cell cycle and oxidative stress markers include ROS production and MDA and GSH-PX activities, suggesting their significant contribution to the distinct cytotoxicity induced by HBCD diastereoisomers. More importantly, given that damaged and dysfunctional mitochondria are responsible for generating most ROS and releasing cytochrome C, leading to apoptosis [37], we also measured the expression of mitochondria-mediated caspases with the treatment of β-HBCD and γ-HBCD in N2a cells. The different regulation pattern of β-HBCD and γ-HBCD in caspase expression demonstrated the important role of mitochondrial apoptotic pathway in HBCD-induced cell apoptosis. Therefore, our work revealed the diastereoisomer-depended cytotoxicity in N2a cells and explicated a potential mechanism involved, which may provide new insights into HBCDs-induced neurotoxicity and may contribute to sophisticated regulatory actions to alleviate their negative impacts on human health.

In this study, we compared the toxic effects of HBCD diastereoisomers on mouse neuroblastoma N2a cells and investigated their potential molecular mechanisms. Among the three diastereoisomers of HBCDs, β-HBCD showed the highest inhibitory effect on cell viability. However, the cytotoxicity of γ-HBCD in N2a cells could not be detected even at maximum solubility, indicating that γ-HBCD did not reach biologically significant toxicity at the tested concentrations. It was reported that the toxicity of HBCDs depended on diastereoisomer selection in vitro. Our result is similar to Shi’s report that the toxicities of the HBCD were in the order of β-HBCD > γ-HBCD > α-HBCD in SH-SY5Y cells [38], another human neuroblastoma cells. Moreover, the cytotoxicity of HBCD diastereoisomers was in the order of β-HBCD ≥ γ-HBCD > α-HBCD in both immortalized human liver cells (L02) and hepatoma cells (HepG2) proved by a series of experiments of cell viability, ROS levels, and DNA damage [28]. The above studies showed that β-HBCD was more toxic than α-HBCD and the results were similar to ours, although the cells used in the studies mentioned above were different. However, the present study found that γ-HBCD exposure does not cause detectable cytotoxicity in N2a cells. This result did not follow previous reports, possibly due to the different metabolism capacities of the three diastereoisomers of HBCD dependent on cell type and species. It has been reported that the metabolic rate of γ-HBCD was higher than that of α-HBCD, and the rank order of total metabolism was still β-HBCD > γ-HBCD > α-HBCD, whether in 34 human milk samples [39], male rats [8], or HepG2 cells and L02 cells [28]. Some of the intermediate metabolites of HBCDs might exert more significant toxic effects than the original compound, which correlated with the origin, occurrence, toxicity, and isomer- or enantiomer-selective enrichment of HBCD diastereomers [40,41].

Furthermore, a series of indicators for oxidation damage induced by HBCDs were examined in the present study. The results showed that the content of MDA significantly increased and the activity of GSH-PX significantly decreased in N2a cells after exposure to α-HBCD and β-HBCD. Thus, both α-HBCD and β-HBCD can induce significant oxidative damage and reduce anti-oxidation ability in N2a cells. The findings verified Zhang’s report on oxidative stress, being an important mechanism in HBCD-mediated cytotoxic effects on HepG2 cells [42]. Furthermore, the levels of mOGG1 at mRNA and protein in N2a cells were upregulated, which indicated that DNA repair was initiated to compensate for the oxidative stress damage by increasing OGG1 expression in N2a cells. Oxidative stress is mainly attributed to ROS [42], which can cause oxidative damage to lipids, proteins, and DNA. Furthermore, DNA lesions need to be repaired to maintain genome integrity [43]. In this study, α-HBCD and β-HBCD induced N2a cells to produce ROS and cause oxidative stress, and MDA level exhibited positive linear correlations with the levels of ROS (*p* < 0.05). This finding is consistent with Shi’s report that the intracellular ROS levels were elevated in HBCD-treated SH-SY5Y cells [38]. Similarly, Zhang et al. [42,44] investigated the cytotoxicity of HBCD enantiomers in HepG2 cells and found that the production of ROS was positively correlated with lactate dehydrogenase release, another biomarker for cell membrane integrity. There are several reasons that may contribute to ROS generation induced by HBCDs. For example, mitochondria serve as the main source of ROS in cells, mitochondrial dysfunction induced by HBCD as observed in this study resulted in the overproduction of ROS [45]. HBCD has been reported to impair the function of the cellular antioxidant system responsible for the neutralization of ROS, thereby increasing ROS production [46]. Consistently, GSH-PX activity, an important cellular antioxidant enzyme that is found in the cytoplasm and mitochondria of mammalian cells, was significantly decreased by both α-HBCD and β-HBCD in our study. Therefore, our data suggested that ROS generation is mainly due to mitochondrial dysfunction and antioxidant system disruption induced by HBCDs.

Excess ROS can cause oxidative damage to the mitochondrial membrane, further inducing mitochondrial dysfunction and apoptosis [47]. The significant enzymatic feature of apoptosis is the activation of caspases. The mitochondria-mediated caspase activation pathway is a major apoptotic pathway [48]. The activation of caspases is tightly controlled by Bcl-2 family proteins, which act primarily by regulating the release of caspase activators in mitochondria. Bcl-2 can inhibit cell apoptosis by maintaining mitochondrial membrane integrity and preventing the release of cytochrome C [49,50]. Caspase-9 is activated and combined with Cyt C, apoptotic protease activating factor1 (Apaf-1), to form an apoptosis-promoting complex [51]. Meanwhile, it is cleaved into cleaved caspase-9, which further activates the downstream apoptotic actuator caspase-3, followed by cleaved caspase-3, which triggers the caspase cascade, leading to cell apoptosis. In this study, HBCD decreased the protein levels of Bcl-2, which shows that the regulation of apoptosis by Bcl-2 on the mitochondrial pathway is weakened, and the release of cytochrome C increases, then leads to an increase in the tendency of cell apoptosis. Moreover, ROS has been shown to regulate caspase activation [52]. We found that HBCDs increased the protein levels of caspase-3, cleaved caspase-3, caspase-9, and cleaved caspase-9, accompanied by increased ROS. These results demonstrate that the mitochondrial apoptosis pathway may be the mechanism of cellular apoptosis induced by HBCDs in an ROS-dependent manner.

It is worth noting that the increase in pro-apoptotic proteins (caspase-3, caspase-9, cleaved caspase-3, and cleaved caspase-9) induced by α-HBCD is greater than caused by β-HBCD, and the decrease in anti-apoptotic protein Bcl-2 caused by α-HBCD was greater than that induced by β-HBCD. Although β-HBCD is less sensitive to apoptosis-related protein activation than α-HBCD, it has greater cytotoxicity than α-HBCD, indicating that other factors may block the events that occur after the initial activation of apoptosis-related proteins. Shemorry et al. [53] showed that caspase-3 could directly establish negative feedback loops generating pro-survival molecules by cleaving specific substrate, which may also be one of the reasons why the cytotoxicity of α-HBCD to N2a is lower than that of β-HBCD. The regulatory mechanism of the activating cell survival and/or apoptotic signaling by α-HBCD and β-HBCD needs further research.

Some studies have found that apoptosis can be induced during cell cycle arrest [54,55]. In this study, we found that cell proliferation was inhibited and the cycle was blocked at the G2/M phase after N2a cells were exposed to α- and β-HBCD. Cell cycle checkpoints are crucial in ensuring that cells have adequate time for DNA repair. Meanwhile, disturbances in the cell cycle control are known to inhibit cell growth and activate apoptosis processes [56]. Thus, excessive ROS induced by HBCDs in N2a cells damages mitochondria and promotes the release of caspase-3 and caspase-9, leading to cell apoptosis, eventually exerting its cytotoxic effects. The findings were similar to those in Shi’s [38] report, in which cell cycle arrest in the sub-G1 phase was induced by HBCD in SH-SY5Y human neuroblastoma cells. Taken together, our results suggest that oxidative stress and cell cycle disruption by the mitochondrial pathway contributed to cytotoxicity in N2a cells induced by HBCDs.

However, our study is limited, as the data were obtained in a cell line. Although mouse neuroblastoma N2a cell lines have been widely used to investigate the neurotoxicity of endocrine-disrupting chemicals [57,58,59], they cannot fully reflect the actual condition of an organism. In particular, in light of the prevalence of multisystemic damage to the organism by endocrine disrupting chemicals, more animal models should be employed to evaluate the neurotoxicity of HBCDs. Therefore, scientifically designed animal models of neurological impairment, indicators of thyroid functions, and more neurological impairment-related assays with HBCD diastereoisomer exposure should be included in future work.

## 5. Conclusions

HBCDs induced diastereoisomers-dependent cytotoxicity to mouse neuroblastoma N2a cells, and the order of cytotoxicity of the three isomers was β-HBCD > α-HBCD > γ-HBCD. Exposure to α-HBCD and β-HBCD resulted in significant oxidative stress and cell cycle disruption, suggesting that oxidative stress, especially elevated ROS levels, probably contributes to apoptosis in N2a cells. Moreover, α-HBCD and β-HBCD differentially increased the mRNA and protein levels of mitochondria apoptosis-related proteins, accompanied by a decrease in the protein levels of Bcl-2 simultaneously. These results demonstrated that the mitochondrial apoptosis pathway may be vital in HBCDs-mediated N2a cell toxicity. Our results highlight the distinct toxic effect of HBCD diastereoisomers, with oxidative stress and alterations in mitochondrial apoptotic pathways suggested as a vital mechanism. Therefore, our findings provide new perspectives for elucidating the diverse neurotoxicity of HBCD diastereoisomers.

## Figures and Tables

**Table 1:** The primer sequences for each mRNA.

Primer Name	Primers Sequence (5′→ 3′)
mOGG1	F: GCATCGTACTCTAGCCTCCAC
	R: CCTCCGTCTGAGTCAGTGTCC
Caspase-3	F: CTGACTGGAAAGCCGAAACTC
	R: CGACCCGTCCTTTGAATTTCT
Caspase-9	F: GGCTGTTAAACCCCTAGACCA
	R: TGACGGGTCCAGCTTCACTA
Bcl-2	F: GTCGCTACCGTCGTGACTTC
	R: CAGACATGCACCTACCCAGC

**Table 2:** Effects on MDA and GSH-PX on N2a cell exposure to HBCD ($\bar{x}$ ± *s*, *n* = 3).

Diastereomers	Chemical Dose (μM)	MDA (nmol/mg Protein)	GSH (U/mg Protein)
α-HBCD	Control	5.19 ± 0.19	196.71 ± 14.26
	1	5.63 ± 0.81	184.88 ± 4.72
	10	8.62 ± 0.38 **	137.46 ± 29.17 **
	50	11.34 ± 1.22 **	109.33 ± 9.01 **
β-HBCD	Control	3.67 ± 0.87	205.50 ± 58.40
	1	3.68 ± 1.06	207.33 ± 57.29
	5	7.55 ± 2.11 *	116.75 ± 14.43 *
	10	9.69 ± 2.88 **	86.89 ± 28.21 *

Note: ** *p* < 0.01; * *p* < 0.05 versus the control.

**Table 3:** Effects on the cell cycle on N2a cell exposure to HBCD ($\bar{x}$ ± *s*, *n* = 3).

Diastereomers	Chemical Dose (μM)	G0/G1 (%)	S (%)	G2/M (%)	G2 + S (%)
α-HBCD	Control	59.2 ± 4.6	33.2 ± 5.6	7.6 ± 1.5	40.8 ± 4.9
	1	60.7 ± 2.5	31.0 ± 3.2	8.4 ± 1.0	39.3 ± 2.5
	10	71.8 ± 4.67 **	12.0 ± 5.3 **	16.3 ± 5.0	28.2 ± 4.7 *
	50	41.0 ± 6. 4 **	32.8 ± 3.2	26.3 ± 6.1	59.0 ± 6.4 **
β-HBCD	Control	63.2 ± 3.7	32.3 ± 4.2	4.5 ± 3.9	36.8 ± 3.7
	1	64.5 ± 4.9	30.4 ± 5.2	5.1 ± 4.3	35.5 ± 4.9
	5	62.0 ± 6.5	18.2 ± 4.6 **	19.8 ± 5.5 **	38.0 ± 6.5
	10	36.0 ± 5.0 **	29.8 ± 1.8	30.0 ± 3.7 **	59.8 ± 5.1 **

Note: ** *p* < 0.01; * *p* < 0.05 versus the control.

## Data Availability

The authors confirm that the data supporting the findings of this study are available within the article [and/or] its Supplementary Materials.

## Supplementary Materials

The following supporting information can be downloaded at: https://kdocs.cn/l/csElucDlsWVr, accessed on 8 September 2024. Figure S1: The complete, unmodified western blots for Bcl-2, cleaved caspases-3 and cleaved caspases-9; Figure S2: Effects on the cell cycle on N2a cell exposure to HBCD.
